# Synthesis and preclinical evaluation of gastrin releasing peptide receptor antagonist [^18^F]MeTz-PEG_2_-RM26 for positron emission tomography

**DOI:** 10.1186/s41181-025-00336-9

**Published:** 2025-03-26

**Authors:** Panagiotis Kanellopoulos, Fanny Lundmark, Ayman Abouzayed, Lorenzo Jacopo Ilic Balestri, Esther Olaniran Håkansson, Karim Obeid, Luke R. Odell, Vladimir Tolmachev, Ulrika Rosenström, Jonas Eriksson, Anna Orlova

**Affiliations:** 1 Department of Medicinal Chemistry, Uppsala University, Uppsala, Sweden; 2 Department of Immunology, Genetics and Pathology, Uppsala University, Uppsala, Sweden; 3 PET Centre, Uppsala University Hospital, Uppsala, 751 85 Sweden; 4 Science for Life Laboratory, Uppsala University, Uppsala, 752 37 Sweden

**Keywords:** Fluorine-18, TCO, Tetrazine, IEDDA click chemistry, GRPR, Bombesin, PET, Prostate cancer

## Abstract

**Background:**

The gastrin-releasing peptide receptor (GRPR) is overexpressed in the majority of primary prostate cancer lesions, with persistent expression in lymph nodes and bone metastases, making it a legitimate molecular target for diagnostic imaging and staging. This study presents the synthesis and preclinical evaluation of [^18^F]MeTz-PEG_2_-RM26, a GRPR antagonist which utilises the Inverse Electron Demand Diels-Alder (IEDDA) reaction for ^18^F-labelling. This click-chemistry approach allows for site-specific incorporation of fluorine-18 under mild conditions, preserving the peptide’s structural integrity and biological activity. Receptor specificity and affinity of [^18^F]MeTz-PEG_2_-RM26 were evaluated in vitro using GRPR-expressing PC-3 cells. Furthermore, the biodistribution profile of [^18^F]MeTz-PEG_2_-RM26 was assessed in NMRI mice and its tumour-targeting capability was investigated in mice bearing PC-3 xenografts.

**Results:**

The labelling of TCO-PEG_2_-RM26 precursor involved three steps: (1) synthesis of an ^18^F-labelled activated ester on a quaternary methyl ammonium (QMA) cartridge, (2) conjugation of the labelled ester to a tetrazine amine, and (3) attachment to TCO-PEG_2_-RM26 via an IEDDA click reaction. This production method of [^18^F]MeTz-PEG_2_-RM26 afforded a high apparent molar activity of 3.5–4.3 GBq/µmol and radiochemical purity exceeding 98%, with 43–70 MBq activity incorporation, while the entire synthesis was completed within 75 min. Both in vitro and in vivo studies confirmed the specific binding of [^18^F]MeTz-PEG_2_-RM26 to GRPR, with a significant reduction in activity uptake observed upon receptor saturation. The radioligand exhibited rapid blood clearance and minimal bone uptake, confirming the stability of the fluorine-carbon bond. However, high hepatic uptake (12–13% IA/g at 1 h post-injection) indicated predominant hepatobiliary excretion. Receptor-mediated uptake was observed in the tumours and pancreatic tissue, although the overall activity uptake in tumours was low, likely due to the rapid hepatobiliary clearance of [^18^F]MeTz-PEG_2_-RM26.

**Conclusions:**

These findings demonstrate the effectiveness of the IEDDA click reaction for fluorine-18 labelling of GRPR-targeting PET tracers. Future studies should focus on increasing the hydrophilicity of the imaging probe to improve the targeting properties and biodistribution profile of the radioligand.

**Supplementary Information:**

The online version contains supplementary material available at 10.1186/s41181-025-00336-9.

## Background

Positron Emission Tomography (PET) is a powerful medical imaging modality that visualises the distribution of tracers labelled with positron-emitting radioisotopes. Widely employed in both biomedical research and clinical practice, PET is used to study biological processes in vivo and diagnose conditions such as cancer, neurological disorders, and cardiovascular diseases. The most commonly used PET tracer is fluorodeoxyglucose ([^18^F]FDG), a glucose analogue labelled with the positron-emitting radioisotope fluorine-18, which has a physical half-life of 110 min. Beyond small molecules, PET imaging also extends to larger biomolecules like peptides and proteins. These biomolecules often exhibit high affinity and selectivity for specific receptors or enzymes along with rapid blood clearance, leading to high-contrast images shortly after administration. In addition to fluorine-18, another common PET radioisotope is gallium-68 with a physical half-life of 68 min. Gallium-68 labelled peptides have proven valuable for precise tumour imaging and receptor characterisation. However, their utility is somewhat constrained by the shorter half-life and the limitations of generator-based production. Therefore, fluorine-18 labelling could be preferred due to its high-yielding radionuclide production and lower positron energy. The half-life of fluorine-18 is also well-aligned with the biological half-life and pharmacokinetic properties of peptides (Zhang et al. 2004). However, the incorporation of fluorine-18 into targeting ligands and biomolecules can pose significant challenges. Traditional radiolabelling methods, such as nucleophilic substitution with [^18^F]fluoride, often require harsh reaction conditions that are unsuitable for peptides and proteins (Jacobson et al. 2015).

To address these challenges, alternative approaches have been developed, including the use of prosthetic groups like [^18^F]fluorobenzaldehyde and *N*-succinimidyl-4-[^18^F]fluorobenzoate ([^18^F]SFB), which provide more controlled labelling processes and have been extensively used to form stable oxime and amide bonds with peptides (Li et al. 2021). Chelation strategies have recently gained traction for fluorine-18 labelling due to the development of chelates allowing aluminium [^18^F]fluoride to be incorporated at low temperatures (Cleeren et al. 2018; Mcbride et al. 2009; Wegrzyniak et al. 2024). Despite this progress, concerns about complex stability remain in certain applications (Archibald and Allott 2021). In addition to these methods, click chemistry has emerged as an increasingly important strategy in PET radiochemistry and fluorine-18 labelling. The copper(I)-catalysed azide-alkyne cycloaddition (CuAAC) has been successfully employed for the high-yield labelling of peptides (Gill and Marik 2011; Li et al. 2017). Another promising click chemistry approach is the Inverse Electron-Demand Diels-Alder (IEDDA) reaction, which offers distinct advantages over copper-catalysed reactions for bioconjugation and labelling of biologically active peptides. The IEDDA click reaction can, for instance, take place between an ^18^F-labelled tetrazine ([^18^F]Tz) and a trans-cyclooctene (TCO) functionalised moiety, providing a versatile and efficient method for fluorine-18 labelling. This bioorthogonal chemistry enables the site-specific incorporation of fluorine-18 into various molecules under mild conditions, including physiological media, without the need for a metal-catalyst (Cheung et al. 2023; Schlein et al. 2024; Syvänen et al. 2020; Wegrzyniak et al. 2023). This approach holds great promise for advancing the development of next-generation PET imaging agents. The diagnostic accuracy could be improved due to combination of the favourable imaging properties of fluorine-18 and the specific biological targeting capabilities of proteins and peptides. Various techniques and methods have been applied to label gastrin releasing peptide receptor (GRPR)-targeting bombesin (BBN) analogues with fluorine-18 (Baratto et al. 2017). Besides conventional nucleophilic substitution with [^18^F]fluoride, which is feasible for compounds with suitable leaving groups in activated positions, such as 4-(*N,N*,*N*-trimethylammonium)benzoate triflate (AlJammaz et al. 2014) or aromatic rings activated with electron-withdrawing groups (Becaud et al. 2009; Höner et al. 2011), alternative strategies include isotopic exchange reactions. This can be performed on di-*tert*-butylfluorosilanes or ammoniomethyl-trifluoroborates, allowing labelling under milder conditions (Dialer et al. 2013). However, labelling of BBN analogues using this method has only been achieved with di-*tert*-butylfluorosilane (Dialer et al. 2013; Pourghiasian et al. 2015; Schirrmacher et al. 2007). Successful labelling of BBN analogues has also been achieved using ^18^F-labelled prosthetic groups, e.g. with *N*-succinimidyl-4-[^18^F]fluorobenzoate, 4-nitrophenyl 2-[^18^F]fluoropropionate, and [^18^F]FDG derivates (Richter et al. 2013, 2015; Yang et al. 2011; Zhang et al. 2006). A disadvantage with these methods is that the starting material cannot be easily separated from the labelled target compound, which might result in a low molar activity due to the need for a relatively high concentration of the peptide. Previous research has shown the antagonistic GRPR-targeting BBN-analogue PEG_2_-RM26 (PEG_2_-D-Phe-Gln-Trp-Ala-Val-Gly-His-Sta-Leu-NH_2_) can be effectively labelled with various radionuclides for use in both single-photon emission computed tomography (SPECT) and PET diagnostics, as well as in radiotherapy (Mitran et al. 2020). This includes the radiolabelling of PEG_2_-RM26 using aluminium [^18^F]fluoride chemistry via NOTA chelator in high radiochemical yield and purity (Varasteh et al. 2013a). However, the method required 2.5 times more peptide than is typically necessary for the chelation of radiometals (Varasteh et al. 2013b). Additionally, sensitivity to radiolysis has been observed during high molar activity labelling of DOTAGA-PEG_2_-RM26 for experimental therapy and the addition of ascorbic acid was needed to preserve the biological properties of the peptide (Mitran et al. 2019).

**Scheme 1:**
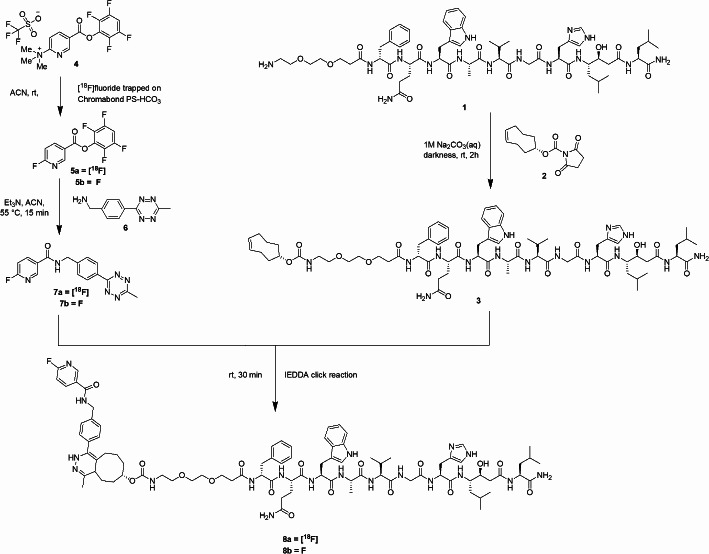
Schematic overview of the synthesis of [^18^F]MeTz-PEG_2_-RM26 (**8a**) and F-MeTz-PEG2-RM26 (**8b**)

As previously mentioned, the CuAAC reaction is a highly efficient click chemistry method for coupling prosthetic groups with peptides under mild conditions. However, its reliance on a copper catalyst limits its application in biological systems due to the cytotoxicity of copper. To address this, copper-free click chemistry reactions, such as strain-promoted alkyne-azide cycloaddition (SPAAC) and ring-strained norbornene-Tz IEDDA cycloaddition, have been investigated for the fluorine-18 labelling of BBN analogues (Campbell-Verduyn et al. 2011; Knight et al. 2013; Murrell et al. 2018). Still, these BBN-based peptides have not yet been evaluated in vivo. The Tz-TCO IEDDA click reaction has successfully incorporated DOTA/DOTAGA-containing prosthetic groups into BBN analogues and it has also been explored as a pre-targeting strategy (D’Onofrio et al. 2021). Recent studies have shown that this reaction achieves quantitative coupling in aqueous solutions and at physiological temperatures (D’Onofrio et al. 2022). Building on these findings, we proposed utilising the Tz-TCO IEDDA click reaction to efficiently radiolabel the GRPR-targeting antagonist PEG_2_-RM26 with fluorine-18 under copper-free conditions and to evaluate the resulting radioligand [^18^F]MeTz-PEG_2_-RM26 **8a** (Scheme 1) in preclinical in vivo studies.

## Methods

### General

All starting materials and solvents were purchased from Sigma Aldrich (MO, USA), Fisher Scientific (PA, USA), MedChemexpress MCE (NJ, USA), Ambeed (Il, USA), Honeywell (NC, USA), Apollo Scientific (SK, UK) and ChemScene (NJ, USA), and used without further purification if nothing else is stated. Analytical high-performance liquid chromatography (HPLC): Dionex UltiMate 3000 HPLC system using Bruker Amazon SL ion trap mass spectrometer and UV detection (diode array detector, 214, 254, and 280 nm). Electrospray ionisation (ESI) MS using a Phenomenex Kinetex C18 column (50 × 3.0 mm, 2.6 μm particle size, 100 Å pore size) with H_2_O/CH_3_CN/0.05% HCOOH (5-100% CH_3_CN) as mobile phase at a flow rate of 1.5 mL/min for 3–5 min. Preparative reversed-phase high-performance liquid chromatography (RP-HPLC): UV-trigged (214 nm) fraction collection with a Gilson HPLC system with a Macherey-Nagel NUCLEODUR C18 HTec column (21 × 125 mm, particle size 5 μm). Silica gel chromatography was carried out on silica gel (Sigma Aldrich, MO, U.S.A), 60 Å pore size, particle size 40–63 nm packed in glass columns. ^1^H NMR and ^13^C NMR were recorded on a Bruker Avance III HD spectrometer at 400 MHz and 101 MHz respectively. The chemical shifts (*δ*) for ^1^H NMR and ^13^C NMR were referenced to tetramethylsilane via residual solvent signals (^1^H: CDCl_3_ at 7.26 ppm; ^13^C{1H}: CDCl_3_ at 77.2 ppm).

The radioactivity measurements were done using a 2480 Wizard2 automatic gamma counter (PerkinElmer, Waltham, MA, USA). The semi-preparative column used for purification of [^18^F]MeTz was an ACE C18 5 μm (150 × 10 mm) column, and the eluent was a mixture of water, ethanol, and TFA in a 62:38:0.01% ratio, with a flow rate of 5 mL/min. The column eluate was monitored using a UV detector (254 nm) and a radiodetector. The radiochemical purity of [^18^F]MeTz was assessed by analytical HPLC using a Kinetex C18 column (2.6 μm 100 Å, 100 × 3.0 mm, Phenomenex, Broenshoej, Denmark). The eluent was a mixture of water and acetonitrile in a 75:25 ratio, with a flow rate of 0.7 mL/min. The identity of the labelled tetrazine was confirmed by co-injection and matching retention times with an authentic reference standard. The molar activity of [^18^F]MeTz was assessed by determining the tracer concentration using the same analytical HPLC procedure and measuring the activity in a dose calibrator.

PC-3 cells (ATCC, Manassas, VA, USA) were maintained at Rosewell Park Memorial Institute 1640 (RPMI 1640) containing L-glutamine and supplemented with 10% fetal bovine serum and 1% penicillin-streptomycin (100 IU/mL penicillin, 100 µg/mL streptomycin). The cells were cultured at 37 °C and 5% CO_2_ in a humidified atmosphere to 80–90% confluency. For subculturing or collection of the cells a solution of 0.25% Trypsin / EDTA (Biochrom AG, Berlin, Germany).

All animal studies were approved by the Ethics Committee for Animal Research in Uppsala, Sweden following the national legislation on protection of laboratory animals (00473/21).

The syntheses of [^18^F]MeTz-PEG_2_-RM26 (**8a**) and F-MeTz-PEG_2_-RM26 (**8b**) were done according to Scheme 1.

### Synthesis of TCO-PEG_2_-RM26 (3)

The peptide-based precursor H_2_N-PEG_2_-RM26 (**1**) (NH_2_-PEG_2_-D-Phe-Gln-Trp-Ala-Val-Gly-His-Sta-Leu-NH_2_, M_w_: 1271.7 g/mol) was synthesized by standard solid phase peptide synthesis (SPPS) using Fmoc Rink Amide 4-Methylbenzhydrylamine (MBHA) resin with loading 0.69 mmol/g as solid support. All coupling reactions were performed in dimethylformamide (DMF) using a 4-fold excess of Fmoc-protected amino acid, Oxyma Pure, *N*,* N’*-diisopropylcarbodiimide (DIC), and diisopropylethylamine (DIPEA). Fmoc-deprotection was done by treatment of 20% piperidine in DMF. After each coupling reaction, as well as Fmoc deprotection, the resin was washed with DMF. The product NH_2_-PEG_2_-RM26 was cleaved from the resin by treatment of trifluoroacetic acid (TFA)/triisopropylsilane (TiPS)/H_2_O (96:2:2) for 6 h. The crude was precipitated in cold diethyl ether × 3 and purified with RP-HPLC using H_2_O/CH_3_CN/0.1% TFA (35–65% CH_3_CN) as mobile phase at a flow rate of 15 mL/min for 20 min (Suppl. Fig. S1). Fractions were pooled and freeze-dried to yield the desired product as a white solid. Calculated [M + 2 H]^2+^: 636.8. Observed [M + 2 H]^2+^: 636.7 ((Suppl. Fig. S2).

To a solution of compound **1** (12 mg, 9.4 µmol) in 1.5 mL sodium carbonate buffer (1 M, pH 8) was added TCO-NHS ester (**2**) (5.0 mg, 20 µmol) dissolved in 200 µL dimethyl sulfoxide (DMSO). The reaction mixture was protected from light and agitated for 2 h at 600 rpm. The crude was purified using RP-HPLC and fractions were pooled and freeze-dried to yield the desired product as a white solid. Calculated [M + H]^+^ and [M + 2 H]^2+^: 1424.9 and 712.9. Observed [M + H]^+^ and [M + 2 H]^2+^: 1424.8 and 712.8 (Suppl. Fig. S3). The purity of the product was analysed on two different columns (C18 and Biphenyl) at different wavelengths (214, 254, and 280 nm) (Suppl. Fig. S4). The product was dissolved in MilliQ water/EtOH (50:50) and stored at -20 °C before use.

### Synthesis of [^18^F]MeTz (7a)

The labelled intermediate [^18^F]Py-TFP (**5a**) was synthesized on a solid support. Aqueous [^18^F]fluoride (16–20 GBq) was concentrated onto a Chromabond PS-HCO_3_ Shorty cartridge (45 mg, Macherey-Nagel, Düren, Germany) and subsequently reacted with a solution of the precursor *N*,*N*,*N*-trimethyl-5-((2,3,5,6-tetrafluorophenoxy)carbonyl)pyridin-2-aminium trifluoromethanesulfonate (**4**) (10 mg) in acetonitrile (0.8 mL) passed over the cartridge, followed by washing with neat acetonitrile (0.7 mL). Both solutions had a flow rate of approximately 0.4 mL/min. The reaction formed [^18^F]Py-TFP (**5a**) at room temperature, and was then released with the flow into a Teflon tubing acting as a reservoir. The solution in the Teflon tubing was then pushed with air over an Oasis MCX Plus Short cartridge (Waters, Milford, Massachusetts, United States), preconditioned with acetonitrile (3 mL), to remove unreacted cationic precursor **4**. The purified solution containing **5a** continued to a septum-equipped vial (5 mL) containing (4-(6-methyl-1,2,4,5-tetrazin-3-yl)phenyl)methanamine hydrochloride **6** (1.0 mg) and DMSO (0.1 mL). Then, triethylamine (5 µL) in acetonitrile (100 µL) was added and the resulting mixture was heated for 15 min at 55 °C. After dilution with aqueous trifluoroacetic acid (3 mL, 0.1%), the labelled tetrazine was purified by semi-preparative HPLC (retention time 8.5 min). The collected fraction containing the desired product **7a** (1–2 GBq, approx. 2 mL), was neutralized with PBS (0.4 mL) and used for the labelling of peptide **3**. The radiochemical purity of **7a** was assessed by analytical HPLC (retention time 6.6 min) (Suppl. Fig. S5).

### Synthesis of [^18^F]MeTz-PEG_2_-RM26 (8a)

A solution of peptide **3** (10–20 nmol) in ethanol (10–20 µL) was added to an aliquot of the collected HPLC fraction containing **7a** (43–70 MBq, 130–200 µL). The mixture was then allowed to react for 30 min at room temperature to form the desired final product **8a** and completely consume **7a**. The radiochemical purity of **8a** was assessed by analytical HPLC using a Kinetex C18 column (2.6 μm 100 Å, 100 × 3.0 mm, Phenomenex). The eluent was a mixture of water + 0.1% formic acid and acetonitrile in a gradient running from 30 to 100% acetonitrile over 15 min, with a flow rate of 0.7 mL/min. The retention time for **8a** was 8–10 min. The identity of the **8a** was assessed by co-elution with the TCO-conjugated peptide (Suppl. Fig. S6).

The synthesis of F-MeTz-PEG_2_-RM26 (**8b**) was performed according to Schemes 1 and S1 (see Supplementary material).

### Synthesis of (2,3,5,6-tetra fluorophenyl) 6-fluoropyridine-3-carboxylate (5b)

6-Fluoro nicotinic acid (**9**) (134 mg, 0.95 mmol) was dissolved in dioxane (5.58 mL). 2,3,5,6-Tetrafluorophenol (**10**) (158 mg, 0.95 mmol) and *N*-Ethyl-*N*′-(3-dimethylaminopropyl)carbodiimide hydrochloride (183 mg, 0.95 mmol) were added and the reaction was stirred overnight at room temperature. The reaction mixture was diluted with CH_2_Cl_2_ (20–50 mL) and washed twice with saturated solution of NaHCO_3_ (20–50 mL), and finally with saturated solution of NaCl (20–50 mL). The organic phase was concentrated in vacuo. The resulting crude product was purified through flash column chromatography (silica gel, 5% EtOAc in isohexane) as a white-off solid; in 90% yield (250 mg). ^1^H NMR (400 MHz, Chloroform-*d*) *δ* 9.09 (d, *J* = 2.4 Hz, 1H), 8.59–8.55 (m, 1H), 7.15–7.04 (m, 2 H). ^13^C NMR (101 MHz, Chloroform-*d*) *δ* 167.9, 165.4, 160.3, 151.6 (d, *J* = 17.5 Hz), 147.5-147.2 (m), 145.0-144.7 (m) 143.5 (d, *J* = 9.7 Hz), 121.9 (d, *J* = 4.3 Hz), 110.2 (d, *J* = 38.5 Hz), 103.86 (t, *J* = 22.8 Hz) (Suppl. Fig. S7 and S8).

### Synthesis of 6-fluoro-N-[[4-(6-methyl-1,2,4,5-tetrazin-3-yl)phenyl]methyl]pyridine-3-carboxamide (7b)

A solution of **6** (16.3 mg, 0.06 mmol) in acetonitrile (3.15 mL) was added to a solution of **5b** (10 mg, 0.04 mmol) in acetonitrile 5 mL and the resulting mixture was heated at 55 °C for 3 h. Then reaction mixture was cooled, concentrated in vacuo and then purified through flash column chromatography (silica gel, gradient elution 30–50% EtOAc in isohexane) to afford compound **7b** as a pink solid in 83% yield (17.4 mg). ^1^H NMR (400 MHz, Chloroform-*d*) *δ* 8.66 (d, *J* = 2.6 Hz, 1H), 8.57 (d, *J* = 8.4 Hz, 2 H), 8.32–8.28 (m, 1H), 7.56 (d, *J* = 8.5 Hz, 2 H), 7.03 (dd, *J* = 8.5, 2.9 Hz, 1H), 6.61 (bs, 1H), 4.77 (d, *J* = 5.8 Hz, 2 H), 3.10 (s, 3 H). ^13^C NMR (101 MHz, Chloroform-d) *δ* 167.4, 166.4, 164.5, 163.8, 146.8, 146.6, 142.4, 141.0, 140.9, 131.4, 128.6, 128.4, 110.1, 109.8, 43.9, 21.2 (Suppl. Fig. S9 and S10).

### Synthesis of F-MeTz-PEG_2_-RM26 (8b)

Synthesis of F-MeTz-PEG_2_-RM26 (**8b**) was performed similarly to synthesis of **8a**. Specifically, 6 mg (4.7 µmol) of **1** was reacted with 2.25 mg (9 µmol) of **2** in 1.5 ml of NaHCO_3_ (1 M) to afford **3** as shown in Scheme 1. Next, 9.95 mg (30.7 µmol) of **7b** was reacted with TCO-PEG_2_-RM26 (**3**) in PBS (pH 7), affording **8b** as a pink solid compound with a total yield of 65%.

### Octanol-water distribution coefficient

Octanol-water distribution coefficient (LogD) was determined experimentally by the addition of 500 µL of n-octanol and 500 µL of MilliQ water to an Eppendorf tube. [^18^F]MeTz-TCO-PEG_2_-RM26 (**8a**) (330 pmol, 1.2 MBq) was added and the tube was vortexed for 2 min. The tube was centrifuged for 20 s to separate the phases. Fractions of each phase were collected and the radioactivity content was measured.

### In vitro competitive binding assay; IC_50_ determination

The half maximal inhibitory concentration (IC_50_) was determined for F-MeTz-PEG_2_-RM2 (**8b**), Ga-loaded NOTA-PEG_2_-RM26 (Varasteh et al. 2013b, 2015) conjugate was used as a comparator. An in vitro competitive binding assay was performed using [^125^I]I-Tyr^4^-BBN (Revvity, Sollentuna, Sweden) similar to the procedure previously described (Kanellopoulos et al. 2024). Briefly, 10^5^ cells were incubated the day before. The next day cells were incubated for 5 h at 4°C with 24.6 fmol of [^125^I]I-Tyr^4^-BBN in the presence of increasing concentrations of competitor (0.01–500 nM). After incubation, the cells were collected, and their activity was measured.

### In vitro binding to GRPR and internalization assay

PC-3 cells (1 × 10^6^ cells/well) were seeded on 6-well dishes one day before the experiment. On the day of the experiment, a set of wells was incubated with 1 µM/well of NOTA-PEG_2_-RM26 for 10 min at rt to block GRPR. After incubation, 8–10 nM (1.2–17.5 kBq) of [^18^F]MeTz-PEG_2_-RM26 (**8a**) was added to the pretreated and non-treated wells, and the cells were incubated for 1 h at 37 °C. Thereafter, cells were detached using trypsin-EDTA (ethylenediaminetetraacetic acid), collected, and measured for their radioactivity content. The internalisation assay was done after 1 h of incubation as previously described (Varasteh et al. 2013b).

### In vivo characterisation

[^18^F]MeTz-PEG_2_-RM26 (**8a**) (30 kBq, 40 pmol) was evaluated in NMRI mice to assess the biodistribution in normal organs. A group of NMRI mice (*n* = 3) was additionally co-injected with 5 nmol of NOTA-PEG_2_-RM26 to block GRPR. Mice were euthanised at 30 min post-injection (pi) and the organs of interest were collected, weighed, and measured for their radioactivity content. Further, Balb/c nu/nu mice (4 mice in each group) were used to evaluate the uptake in normal organs and the GRPR-expressing tumour model (PC-3). In short, 7 × 10^6^ freshly harvested PC-3 cells were suspended in 100 µl sterile PBS and were implanted subcutaneously at the right hind leg of each mouse. After 3–4 weeks, well-palpable tumours (130–600 mg) were developed at the implantation sites. [^18^F]MeTz-PEG_2_-RM26 (**8a**) (120 kBq, 48 pmol) was injected and mice were euthanised at predetermined time points of 1 and 2 h pi. An additional group was co-injected with 5 nmol of NOTA-PEG_2_-RM26 to block GRPR and was euthanized at 2 h pi. The collected organs were weighed and the radioactivity content was measured in each organ.

## Results

### Synthesis of [^18^F]MeTz (7a) and [^18^F]MeTz-PEG_2_-RM26 (8a)

The peptide-based precursor H_2_N-PEG_2_-RM26 was successfully synthesized via Fmoc SPPS. After cleavage from the resin, the peptide was conjugated to TCO-NHS ester in a sodium carbonate buffer and purified by RP-HPLC. The fluorine-18 labelled prosthetic group [^18^F]MeTz was successfully synthesized by a three-step method. [^18^F]MeTz (**7a**) was obtained in a radiochemical yield of 18 ± 6%, a radioactivity yield of 2.0 ± 0.5 GBq, and a radiochemical purity of > 98% (*n* = 5) (Fig. S5). The molar activity at the end of synthesis was 190–220 GBq/µmol. Finally, the desired GRPR-targeting radioligand [^18^F]MeTz-PEG_2_-RM26 (**8a**) was obtained by IEDDA click reaction at rt. [^18^F]MeTz-PEG_2_-RM26 was formulated in 32% ethanol in PBS and obtained with a radiochemical conversion yield of > 99% based on [^18^F]MeTz (Fig. S6). The radioactivity yield was 43–70 MBq and the radiochemical purity was > 98% at the end of synthesis. The apparent molar activity of [^18^F]MeTz-PEG_2_-RM26 (**8a**) at the end of synthesis was 3.5–4.3 GBq/µmol (*n* = 5). The cold analogue F-MeTz-PEG_2_-RM26 (**8b**) was prepared in an analogous fashion for comparison.

### In vitro competitive binding assay; IC_50_ determination

**Fig. 1:**
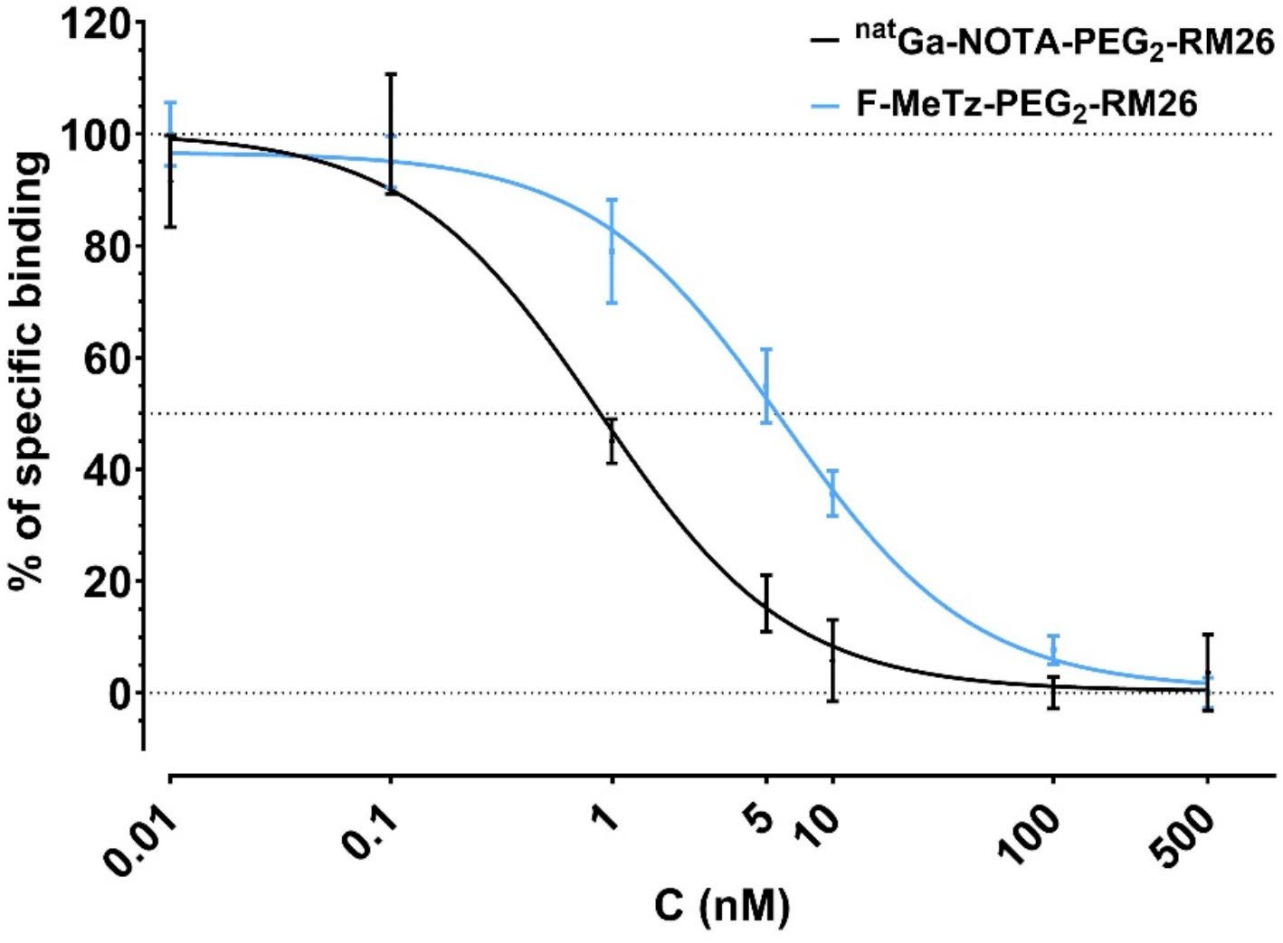
Competition binding curves of F-MeTz-PEG_2_-RM26 (**8b**) and Ga-NOTA-PEG_2_-RM26 against [^125^I]I-Tyr^4^-BBN in alive PC-3 cells. The data represent the mean ± standard deviation

Competition binding assays were performed on PC-3 cells for F-MeTz-PEG_2_-RM26 (**8b**) and Ga-NOTA-PEG_2_-RM26 (reference), against [^125^I]I-Tyr^4^-BBN. Both compounds displayed high GRPR affinity. Specifically, **8b** had an IC_50_ value of 5.9 nM, while the IC_50_ for Ga-NOTA-PEG_2_-RM26 was 0.87 nM. Representative curves are shown in Fig. 1.

### In vitro characterization of [^18^F]MeTz-PEG_2_-RM26 (8a)

**Fig. 2:**
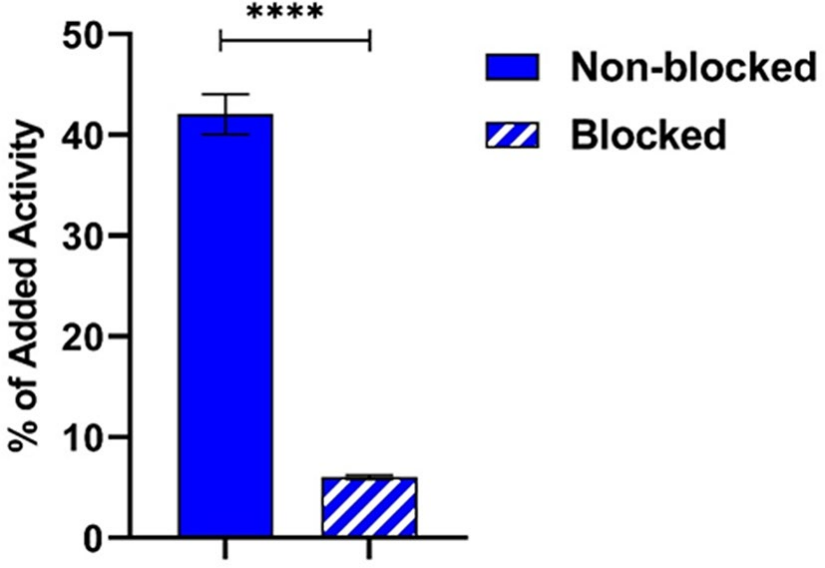
In vitro binding of [^18^F]MeTz-PEG_2_-RM26 (**8a**) to GRPR with and without preblocking of GRPR. The data represent the mean ± standard deviation. **** denoted as P-value < 0.0001, *n* = 3

The in vitro binding of [^18^F]MeTz-PEG_2_-RM26 (**8a**) to GRPR was investigated using GRPR-expressing PC-3 cells. The study demonstrated a statistically significant reduction of activity uptake upon target saturation (as shown in Fig. 2) achieved by pre-treating the cells with an excess of non-labelled NOTA-PEG_2_-RM26.

The results from the internalization assay demonstrated a relatively fast internalization of [^18^F]MeTz-PEG_2_-RM26 (**8a**) for an antagonist. Approximately 25% of the cell-associated activity was found in the internalized fraction at 1 h incubation. The octanol-water distribution coefficient (LogD) of [^18^F]MeTz-PEG_2_-RM26 (**8a**) was 1.6 ± 0.2.

### In vivo characterization of [^18^F]MeTz-PEG_2_-RM26 (8a)

**Fig. 3:**
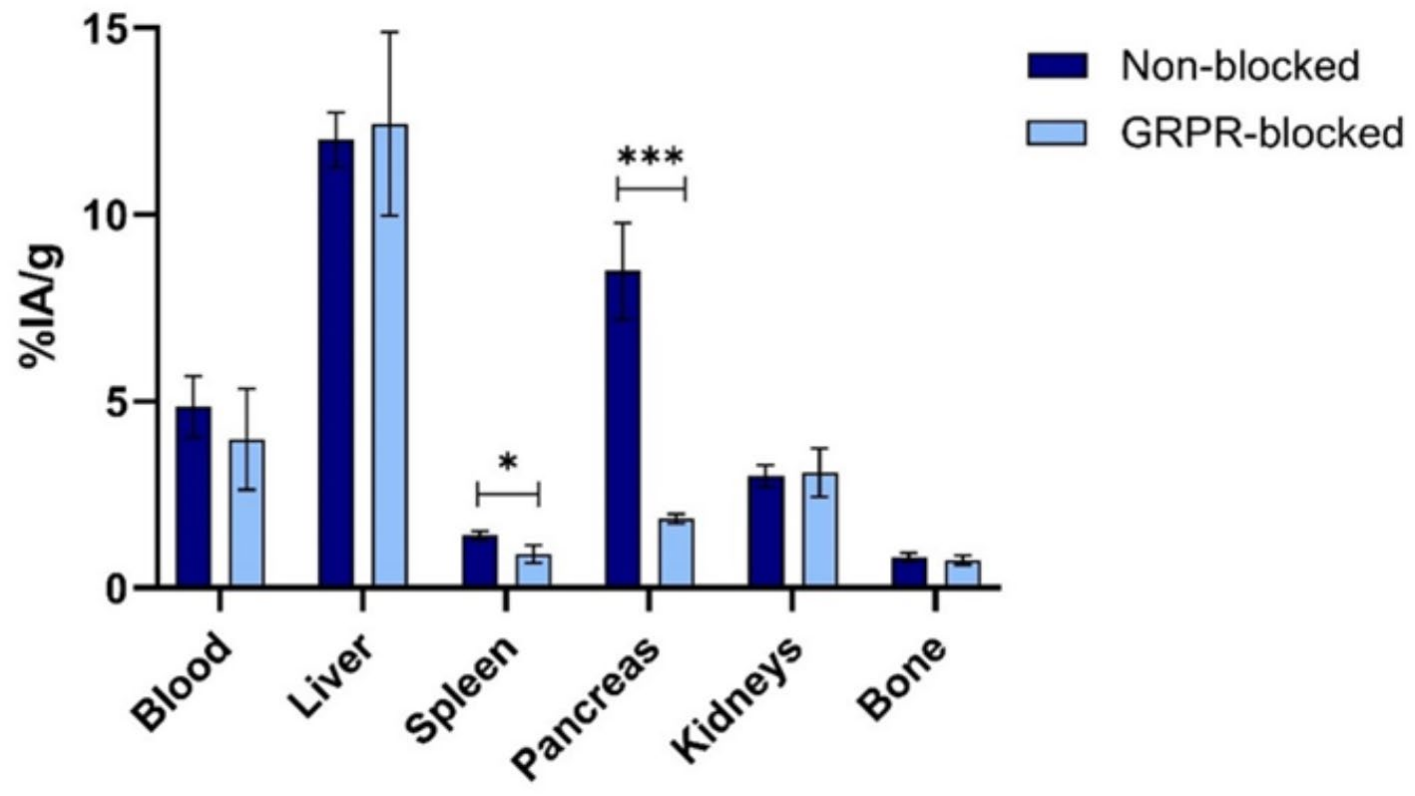
Biodistribution of [^18^F]MeTz-PEG_2_-RM26 (**8a**) in NMRI mice at 30 min pi. A statistically significant difference in activity uptake was found for the spleen (*P* < 0.05) and the pancreas (*P* < 0.0005, *n* = 3–4)

The biodistribution of [^18^F]MeTz-PEG_2_-RM26 (**8a**) in NMRI mice at 30 min post-injection (pi) indicated rapid clearance of the labelled peptide from blood circulation (Fig. 3 and Suppl. Table S1). The activity concentration in blood was 5 ± 1% of injected activity per gram (% IA/g). High activity accumulation was found in the liver (12.0 ± 0.7% IA/g), while the activity uptake in kidneys was four-fold lower (3.0 ± 0.1% IA/g). Low activity uptake was also found in bones (0.8 ± 0.1% IA/g). The activity uptake in the pancreas was significantly lower in the group co-injected with a non-labelled GRPR-blocking agent (1.9 ± 0.6% IA/g) compared to the non-blocked group (9 ± 1% IA/g).

**Fig. 4:**
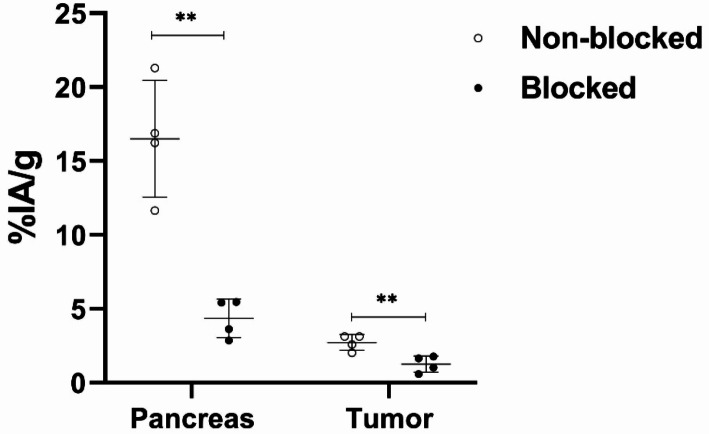
The [^18^F]MeTz-PEG_2_-RM26 (**8a**) in vivo targeting of GRPR-expressing tissue. The data represent the mean ± standard deviation. ** denoted as P-value < 0.01, *n* = 4

The blocking of GRPR in tumour-bearing mice resulted in a significantly reduced activity uptake in the pancreas (4 ± 1% IA/g, *P* < 0.01, *n* = 4) and tumours (1.3 ± 0.6% IA/g, *P* < 0.01, *n* = 4) and the results are shown in Fig. 4 and Suppl. Table S2. There was no significant difference in activity uptake in GRPR-negative normal organs and tissues between non-blocked and blocked groups.

**Fig. 5:**
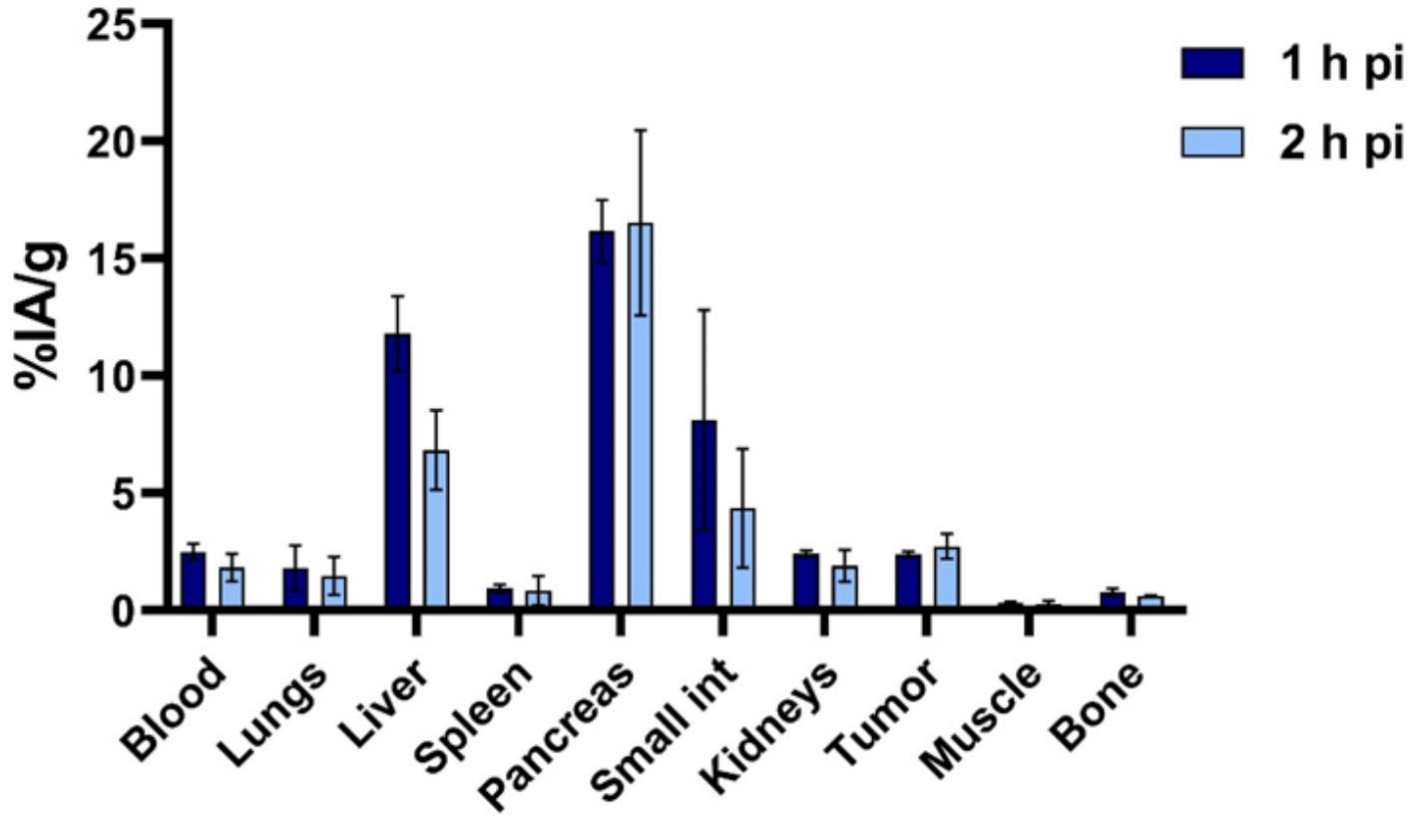
The biodistribution of [^18^F]MeTz-PEG_2_-RM26 (**8a**) in PC-3 tumour-bearing Balb/c nu/nu mice at 1 and 2 h pi. The data represent the mean ± standard deviation, *n* = 4

The biodistribution of [^18^F]MeTz-PEG_2_-RM26 (**8a**) was investigated at 1 and 2 h pi in mice bearing GRPR-positive PC-3 xenografts (Fig. 5 and Suppl. Table S2). The overall distribution pattern of the radioligand in tumour-bearing mice was similar to that observed in NMRI mice. Most GRPR-negative normal organs and tissues demonstrated a general trend of decreasing activity uptake over time, however this was not statistically significant. Notably, the liver exhibited a significant reduction in activity uptake, with levels decreasing by approximately two-fold at 2 h pi compared to 1 h pi (*P* < 0.01, *n* = 4). In contrast, the activity uptake in GRPR-positive pancreatic tissue and xenografts remained consistent throughout the observation period. The tumour uptake was 2.4 ± 0.1 and 2.7 ± 0.5% IA/g at 1 and 2 h pi (*P* > 0.05, *n* = 4).

Tumour-to-organ (T/O) ratios are presented in Table 1. At 1 h pi, values over 1 (positive contrast in imaging) were detected in the lungs, spleen, muscles, and bones. T/O ratios significantly increased in blood and liver at 2 h pi, however, the tumour-to-liver ratio was still below 1. Other investigated organs and tissues, lungs, spleen, pancreas, intestines, kidney, muscle, bones, demonstrated a tendency to increase T/O ratios with time (*P* > 0.05, *n* = 4).

**Table 1:** Tumour-to-non-tumour ratios of [^18^F]MeTz-PEG_2_-RM26 (**8a**) at 1 and 2 h Pi in GRPR-expressing PC-3 tumour-bearing mice. Statistical analysis was performed using a multiple t-test model

	Tumour-to-organ ratio	
Organ	1 h pi.	2 h pi.
Blood	0.97 ± 0.1	1.5 ± 0.2^a^
Lungs	1.9 ± 0.1	2.2 ± 1.0
Liver	0.20 ± 0.02	0.4 ± 0.1^a^
Spleen	2.6 ± 0.5	4.0 ± 2
Pancreas	0.15 ± 0.02	0.17 ± 0.05
Small intestine	0.50 ± 0.2	0.70 ± 0.3
Kidneys	0.99 ± 0.08	1.6 ± 0.6
Muscle	7.4 ± 0.7	12 ± 4
Bone	3.2 ± 0.5	5.0 ± 1^a^

a - statistical difference between 1 h and 2 h pi, *p* < 0.01, *n* = 4

## Discussion

The GRPR-targeting peptide-based antagonist PEG_2_-RM26 was successfully labelled with fluorine-18 using a method originally developed for the labelling of antibody constructs and affibody molecules. The synthesis proceeded in three steps, beginning with the preparation of the activated ester [^18^F]Py-TFP (**5**) on a solid support using a QMA cartridge with trapped [^18^F]fluoride. This approach eliminated the need for conventional azeotropic drying of [^18^F]fluoride and the associated reaction vessel. The labelled ester was then converted to a tetrazine amide (**7a**) via amidation and after semi-preparative HPLC purification, 2.0-2.5 GBq of [^18^F]MeTz (**7a**) was typically obtained. An aliquot of the purified [^18^F]MeTz (**7a**) was subsequently reacted with the TCO-PEG_2_-RM26 (**3**) peptide functionalised with one TCO group. The IEDDA click reaction forming [^18^F]MeTz-PEG_2_-RM26 (**8a**) was completed within 30 min at rt, achieving full incorporation of [^18^F]MeTz (**7a**) into TCO-PEG_2_-RM26 (**3**). The total synthesis time, from the initiation of the [^18^F]MeTz synthesis to the preparation of [^18^F]MeTz-PEG_2_-RM26 (**8a**) for injection, was 75 min. These results confirm the successful use of the Tz-TCO IEDDA click reaction for fluorine-18 labelling of not only larger biomolecules like antibody constructs and affibody molecules, but also for peptides.

Notably, only 10–20 nmol of TCO-PEG_2_-RM26 (**3**) was required for the labelling, achieving a quantitative radiochemical yield by fully consuming all [^18^F]MeTz (**7a**) and producing 43–70 MBq of the radiolabelled peptide — sufficient for biological evaluations. The radiochemical purity exceeded 98%, negating the need for further purification steps (Fig. S6). The apparent molar activity, ranging from 3.5 to 4.3 GBq/µmol, was calculated based on the activity in the product solution and the amount of TCO-PEG_2_-RM26 (**3**) used during the labelling reaction. The term apparent molar activity was used since the peptide precursor molecule was not separated from the labelled compound. This level of molar activity is adequate for clinical application, as studies have shown that 15–30 nmol of a GRPR-targeting antagonist is sufficient to achieve high-contrast imaging (Chernov et al. 2023; Nock et al. 2021; Stoykow et al. 2016; Wieser et al. 2014). Similar to labelling with radiometals using chelation chemistry, it was not feasible to separate [^18^F]MeTz-PEG_2_-RM26 (**8a**) from the unreacted TCO-PEG_2_-RM26 (**3**) starting material. Therefore, minimising the peptide concentration in the reaction was essential for maintaining high apparent molar activity. The ratio of [^18^F]MeTz (**7a**) to the number of available TCO groups was estimated to be between 1:50 and 1:60, highlighting the efficiency of the IEDDA click reaction in facilitating fluorine-18 labelling. This approach enabled a 1000-fold reduction in the required peptide amount compared to conventional fluorine-18 nucleophilic substitution reactions, which typically require precursor quantities at the micromolar scale.

The labelling method presented in this study offers several significant advantages over other fluorination techniques. Notably, it is copper-free, rapid, and efficient, with the labelling procedure occurring at room temperature, yielding a final product that requires no additional purification. For instance, alternative methods for the labelling of BBN analogues typically result in considerably lower yields. The ring-strained norbornene-tetrazine IEDDA click reaction and the SPAAC click reaction generally result in yields between 20 and 45% (Campbell-Verduyn et al. 2011; Knight et al. 2013; Paulus et al. 2015). To achieve higher yields, the temperature of the labelling reaction could be elevated or a copper catalyst added, as in the CuAAC click reaction (Li et al. 2013; Paulus et al. 2015; Sachin et al. 2012). However, increasing the reaction temperature could potentially impair the integrity of certain biomolecules (Murrell et al. 2018), while the use of copper could introduce cytotoxic risks to biological systems. Therefore, these alternatives should be carefully considered based on the specific application and desired outcomes.

The biological activity and integrity of [^18^F]MeTz-PEG2-RM26 (**8a**) were well-preserved following the labelling process, with specific binding to GRPR confirmed both in vitro and in vivo. The direct comparison of the half maximal inhibitory concentration (IC_50_) values for F-MeTz-PEG_2_-RM26 (**8b**) (5.92 nM) and Ga-NOTA-PEG_2_-RM26 (0.87 nM) (Varasteh et al. 2013b) demonstrated a 6.8-fold decrease in binding affinity for the new conjugate. Based on this, we can speculate that the bulky group used for labelling of peptide with fluorine-18 might interfere with the radioligand–receptor recognition and interaction. This also corroborates with the relatively fast internalization observed for [^18^F]MeTz-PEG_2_-RM26 (**8a**). It was reported that antagonist [^68^Ga]Ga-NOTA-PEG_2_-RM26 had an internalisation rate of 8% (from cell associated activity) within 30 min while the agonist [^125^I]I-Tyr^4^-BBN reached 50% (Varasteh et al. 2013b). The internalization rate for [^18^F]MeTz-PEG_2_-RM26 (**8a**) was 25% within 1 h, and is comparable to previous studies. There was no evidence of radiocatabolite efflux associated with the non-residualizing properties of the ^18^F-label, as the internalized fraction of cell-associated activity remained stable over time. Additionally, the biological evaluation showed no significant signs of in vivo defluorination. These findings underscore the potential of this labelling technique in advancing the development of targeted radiopharmaceuticals for clinical use.

The biodistribution of [^18^F]MeTz-PEG_2_-RM26 (**8a**) was assessed at 30 min pi in healthy mice, both with and without co-injection of a non-labelled GRPR-targeting blocking agent. Despite rapid blood clearance, whole-body activity retention was high (84 ± 7% IA). Clearance was primarily hepatic, based on the significant accumulation in the liver (12.0 ± 0.7% IA/g) and fast translocation of radiocatabolites into the intestinal content (38 ± 4% IA per whole intestine with content). The activity uptake in kidneys (3.0 ± 0.1% IA/g) and the observed decrease in whole-body activity within 30 min suggests minor renal excretion. Low activity uptake in bones (0.8 ± 0.1% IA/g) indicated high stability of the fluorine-carbon bond. In the pancreas, activity uptake was 9 ± 1% IA/g in the non-blocked group injected with only [^18^F]MeTz-PEG_2_-RM26 (**8a**), while it was significantly reduced (1.9 ± 0.6% IA/g) in the GRPR-blocked group co-injected with a 100× molar excess of non-labelled NOTA-PEG_2_-RM26. Since the pancreas is a GRPR-positive tissue, this highlights the GRPR specificity and binding capacity of [¹⁸F]-MeTz-PEG₂-RM26 (**8a**). These biodistribution results in NMRI mice support further in vivo characterization of [^18^F]MeTz-PEG_2_-RM26 (**8a**) in mice bearing GRPR-positive PC-3 tumours.

The overall pattern of activity distribution in PC-3 tumour-bearing mice mirrored that observed in NMRI mice, characterized by rapid clearance through the hepatobiliary system and minimal activity uptake in bones. Most GRPR-negative normal organs and tissues exhibited a general decrease in activity uptake over time. Notably, the liver showed a significant reduction in activity uptake with approximately a two-fold decrease between 1 and 2 h pi (*P* < 0.01, *n* = 4). In contrast, activity uptake in GRPR-positive pancreatic tissue and xenografts remained stable throughout the observation period. Although stable, the activity uptake in the tumour was rather low (2.4 ± 0.1% IA/g at 1 h pi and 2.7 ± 0.5% IA/g at 2 h pi.), resulting in a six-fold higher activity uptake in the pancreas compared to the tumours. However, the blocking of GRPR resulted in a significantly reduced activity uptake both in the pancreas (4 ± 1% IA/g) and in tumours (1.3 ± 0.6% IA/g) (Fig. 5). There was no significant difference in activity uptake in any other investigated organs and tissues between the non-blocked and blocked groups. These findings confirm that the activity uptake of [^18^F]MeTz-PEG_2_-RM26 (**8a**) was receptor-mediated both in the pancreas and PC-3 xenografts.

The low activity uptake in tumours was unexpected, especially given the reasonable affinity to GRPR and substantial GRPR-mediated uptake observed in normal pancreatic tissue. However, this phenomenon is similar to the biodistribution pattern observed for other BBN-based ligands labelled with fluorine-18 (Dialer et al. 2013; Höhne et al. 2008; Höner et al. 2011; Li et al. 2013; Mu et al. 2010; Paulus et al. 2015; Richter et al. 2015). All reported ligands demonstrated activity uptake in tumours lower than in pancreas, except the ligand labeled with highly hydrophilic [^18^F]FDG for which activity uptake in pancreas was equal to that in tumours (Richter et al. 2015). Reported studies describe consistently high levels of hepatobiliary excretion, similar to that observed with [^18^F]MeTz-PEG_2_-RM26 (**8a**). The hepatic uptake remained unsaturated even when a high amount of non-labelled GRPR blocking agent was co-injected, suggesting that the lipophilic nature of the radioligand contributed significantly to this off-target interaction. Supporting this, the octanol-water distribution coefficient (LogD) for [^18^F]MeTz-PEG_2_-RM26 (**8a**) was 1.6 ± 0.2 compared to the highly hydrophilic [^68^Ga]Ga-NOTA-PEG_2_-RM26 (-2.27 ± 0.07). We can speculate that a high level of hepatobiliary excretion rapidly decreases the blood concentration of this ligand and subsequently their availability and penetration in xenografts, but not in normally vascularised pancreatic tissue. To address this issue, enhancing the hydrophilicity of the GRPR linker connecting the targeting peptide sequence and the TCO functionality could be a valuable strategy to continue the development of fluorine-18 labelled GRPR-targeting radioligands and improve the targeting properties. In addition to the GRPR-linker, structural modifications to the prosthetic group to decrease its lipophilicity could be another approach for further optimization.

## Conclusions

The successful synthesis of [^18^F]MeTz-PEG_2_-RM26 (**8a**) using the Tz-TCO IEDDA click chemistry reaction has demonstrated a highly efficient, rapid, and chemically mild approach for fluorine-18 labelling of peptides. This labelling technique preserves the biological integrity of the peptide, resulting in high radiochemical purity and apparent molar activity, without any additional purification. The efficient labelling method with several advantages over other fluorine-18 labelling techniques, together with the in vivo specific binding of [^18^F]MeTz-PEG_2_-RM26 (**8a**) to GRPR, highlights its potential as a valuable tool for PET imaging of GRPR expression. This study represents a significant initial step in advancing the development of fluorine-18 labelled radioligands using Tz-TCO IEDDA click chemistry method, aiming to refine the molecular design and enhance physiological and pharmacokinetic properties.

## Electronic supplementary material

Below is the link to the electronic supplementary material.

Supplementary Material 1

## Data Availability

All data generated or analysed during this study are included in this published article and its supplementary information files.

## Abbreviations

BBN: Bombesin
CuAAC: Copper(I)-catalysed azide-alkyne cycloaddition
DMF: Dimethylformamide
DMSO: Dimethyl sulfoxide
IC_50_: Half maximal inhibitory concentration
ESI: Electrospray ionisation (MS)
GRPR: Gastrin-releasing peptide receptor
HPLC: High-performance liquid chromatography
IEDDA: Inverse electron demand diels-alder (reaction)
MeTz: Methyl tetrazine
NMRI: Naval Medical Research Institute
PBS: Phosphate buffered saline
PET: Positron emission tomography
Pi: Post-injection
RP-HPLC: Reversed-phase high-performance liquid chromatography
SPAAC: Strain-promoted alkyne-azide cycloaddition
SPPS: Solid phase peptide synthesis
TCO: Trans-cyclooctene
TFA: Trifluoroacetic acid
T/O: Tumour-to-organ (ratios)
% IA/g: % of injected activity per gram
[^18^F]FDG: Fluorodeoxyglucose
