# Supplementary material for: Synthesis and preclinical evaluation of gastrin releasing peptide receptor antagonist [18F]MeTz-PEG2-RM26 for positron emission tomography
Source: EJNMMI Radiopharm Chem. 2025 Mar 26;10:14. doi: 10.1186/s41181-025-00336-9 (PMC11947346; doi:10.1186/s41181-025-00336-9)
Supplement: Supplementary file 1 — Supplementary Material 1 [file 41181_2025_336_MOESM1_ESM.docx]

**Supplementary information**

**Scheme S1**. Schematic overview of the synthesis of F-MeTz-PEG_2_-RM26 (**8b**).


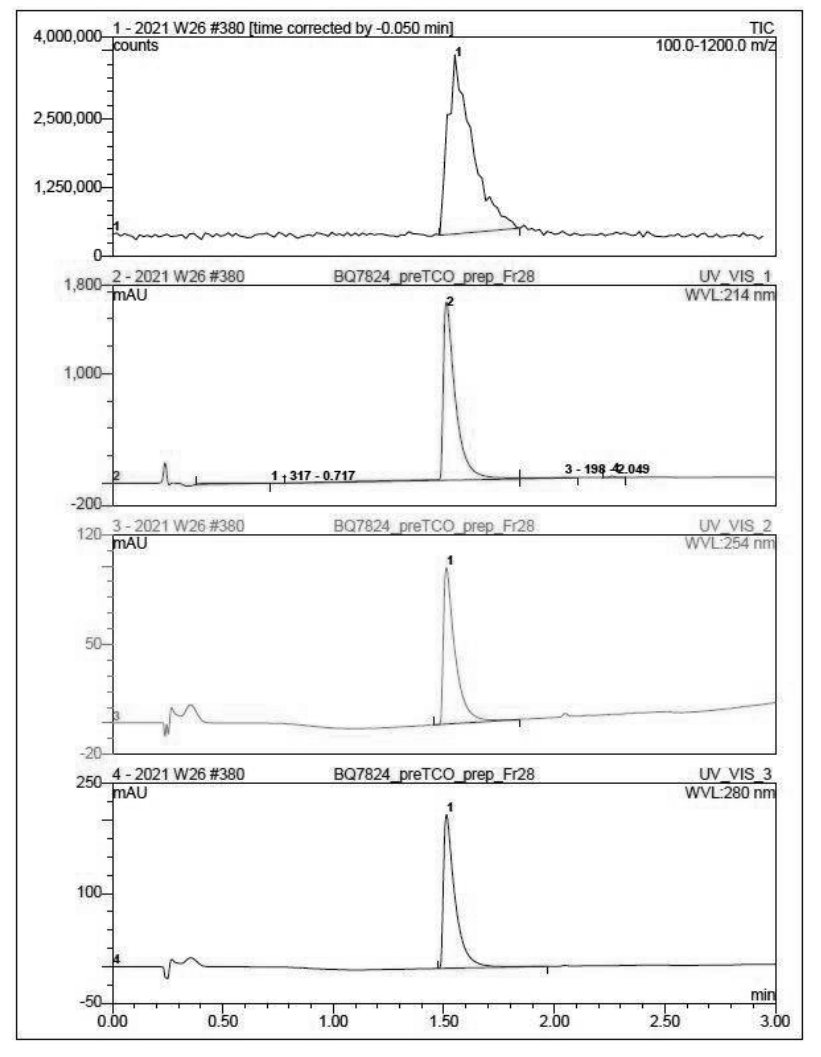


**Figure S1**. Analytical RP-HPLC for **1,** C18 column, gradient 5-100% of 0.05% formic acid in ACN for 3 min. Total ionization chromatogram (TIC) and UV detection at 214, 254, and 280 nm (from top to bottom).


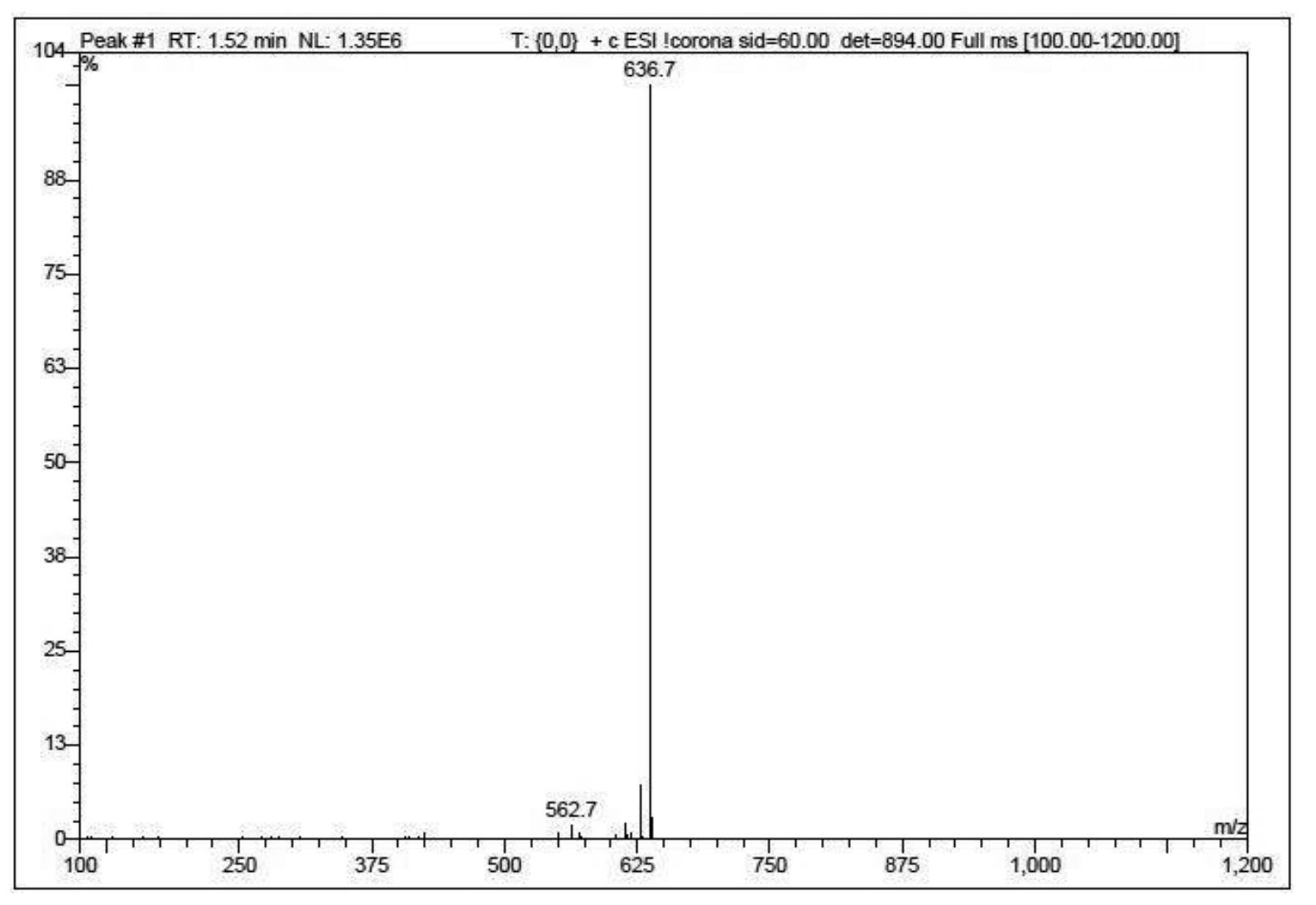


**Figure S2.** Mass spectrometry chromatogram of **1**. Calculated [M+2H]^2+^: 636.85. Observed [M+2H]^2+^: 636.7.


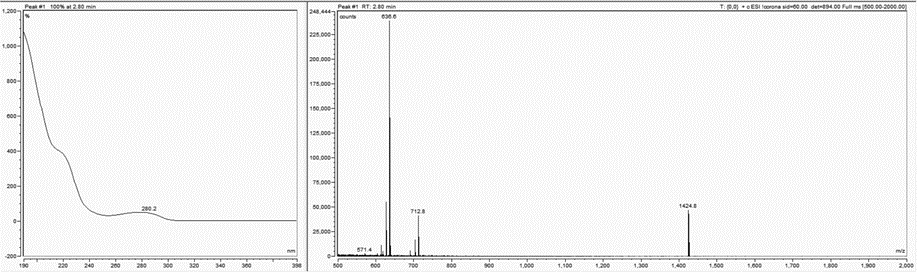


**Figure S3**. Mass spectrometry chromatogram of **3**. Calculated [M+H]+ and [M+2H]2+: 1424.9 and 712.9. Observed [M+H]+ and [M+2H]2+: 1424.8 and 712.8.


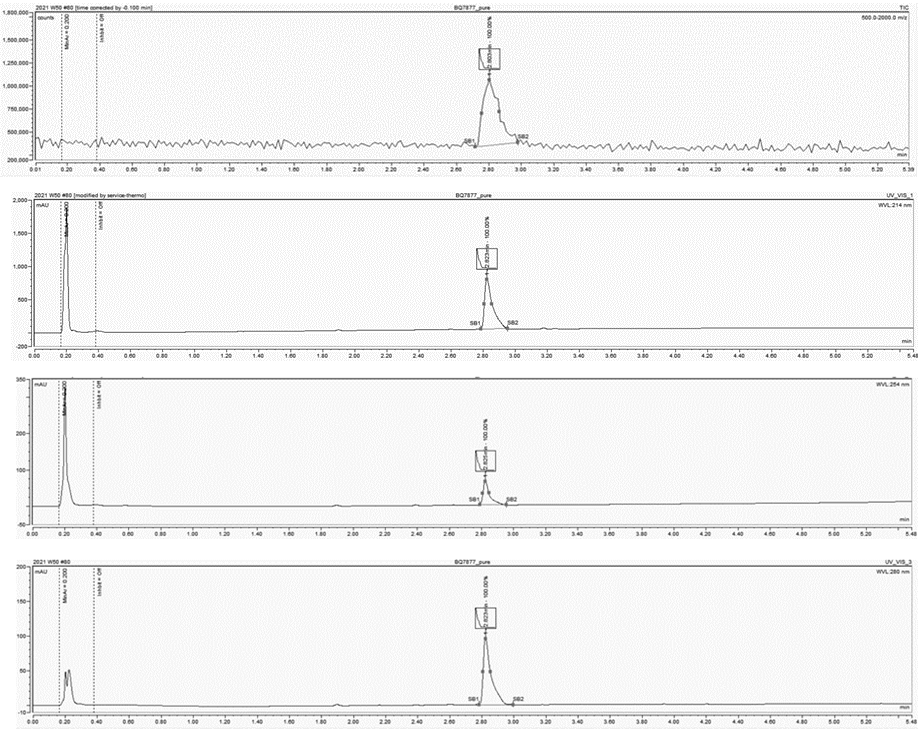


**Figure S4**. Analytical RP-HPLC of **3,** C18 column, gradient 5-100% of 0.05% formic acid in ACN for 3 min. Total ionization chromatogram (TIC) and UV detection at 214, 254, and 280 nm (from top to bottom).


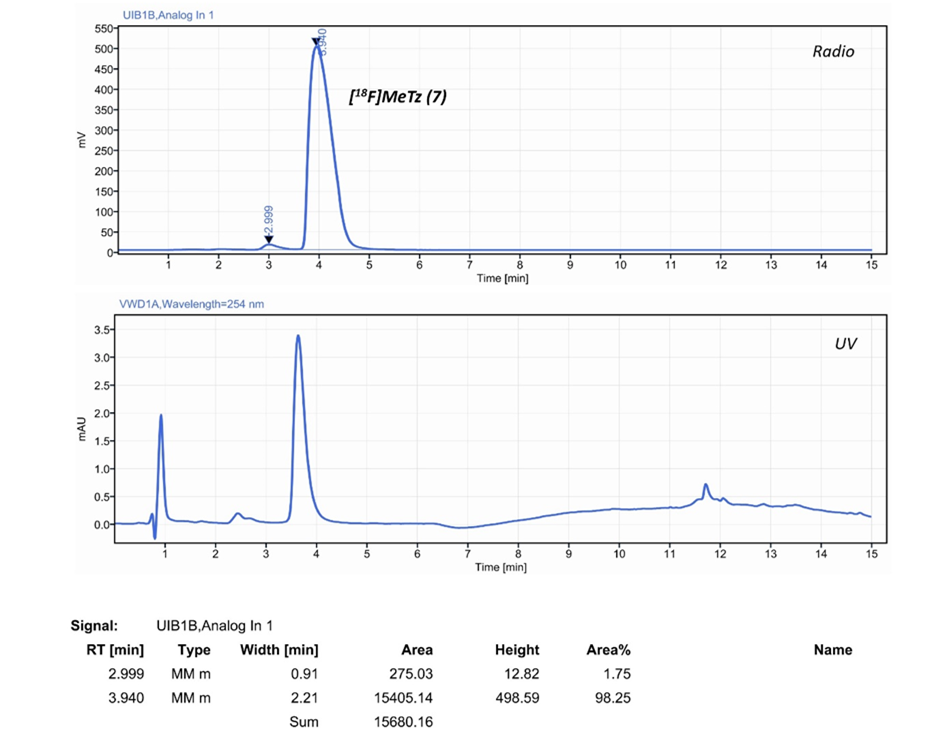


**Figure S5**. Analytical liquid chromatography of **7a**.


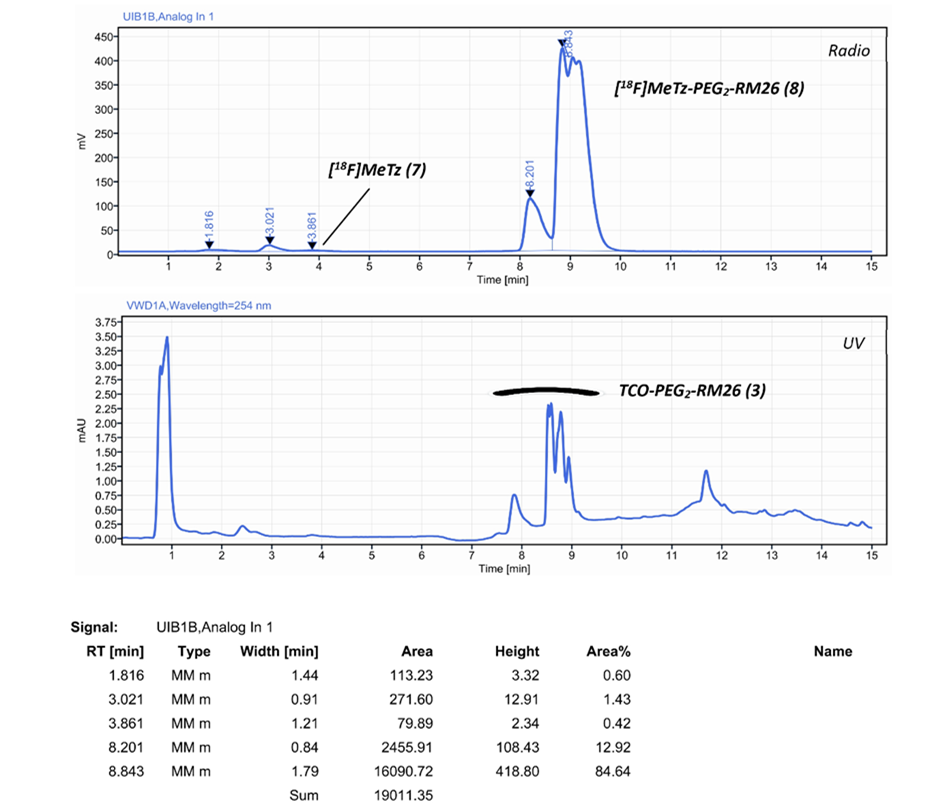


**Figure S6**. Analytical liquid chromatography of **8a**.

**Figure 7**. **^1^H-NMR** (400 MHz, CDCl_3_) spectra of **5b**

**Figure S8**. **^13^C-NMR** (101 MHz, CDCl_3_) spectra of **5b**

**Figure S9**. **^1^H-NMR** (400 MHz, CDCl_3_) spectra of **7b**

**Figure S10**. **^13^C-NMR** (101 MHz, CDCl_3_) spectra of **7b**

**Table S1**. Biodistribution in NMRI 30 min pi of 40 pmol [^18^F]MeTz-TCO-PEG_2_-RM26 (**8a**) (non-blocked). Data are presented as %IA/g, except samples of organs of the gastrointestinal tract with content and the rest of the the carcass that are presented as %IA/sample. * and *** - values are significantly higher for the non-blocked group.

|  | Non-blocked | Blocked |
| --- | --- | --- |
| Blood | 4.9±0.8 | 4±1 |
| Liver | 12.0±0.7 | 12±2 |
| Spleen | 1.4±0.1* | 0.9±0.2 |
| Pancreas | 8±1*** | 1.9±0.1 |
| Kidneys | 3.0±0.3 | 3.1±0.6 |
| Bone | 0.8±0.1 | 0.7±0.1 |
| GI tract | 38±4 | 32±4 |
| Carcas | 15±2 | 16±4 |

**Table S2**. Biodistribution 1 and 2 h pi of 40 pmol [^18^F]MeTz-TCO-PEG_2_-RM26 (**8a**) in PC-3 xenografted mice. Data are presented as %IA/g. * - values are significantly different from the 2 h pi group.

|  | 1 h | 2 h | 2 h blocked |
| --- | --- | --- | --- |
| Blood | 2.5±0.4 | 1.8±0.6 | 2.1±0.3 |
| Lungs | 1.3±0.1 | 1.5±0.8 | 1.0±0.2 |
| Liver | 12±2* | 7±2 | 6±2 |
| Spleen | 0.9±0.2 | 0.8±0.6 | 0.63±0.07 |
| Pancreas | 16±1 | 17±4 | 4±1* |
| Small int | 6±3 | 4±3 | 5±2 |
| Kidneys | 2.4±0.1 | 1.9±0.7 | 1.1±0.2 |
| Tumor | 2.4±0.1 | 2.7±0.5 | 1.3±0.6* |
| Muscle | 0.33±0.04 | 0.3±0.1 | 0.20±0.05 |
| Bone | 0.8±0.2 | 0.60±0.04 | 0.71±0.24 |
